# Cognitive Workload Detection of Air Traffic Controllers Based on mRMR and Fewer EEG Channels

**DOI:** 10.3390/brainsci14080811

**Published:** 2024-08-13

**Authors:** Li Hui, Zhu Pei, Shao Quan, Xue Ke, Sun Zhe

**Affiliations:** College of Civil Aviation, Nanjing University of Aeronautics and Astronautics, Nanjing 211106, China; lihui123@nuaa.edu.cn (L.H.); zpei@nuaa.edu.cn (Z.P.); xue.ke@nuaa.edu.cn (X.K.); sz2207075@nuaa.edu.cn (S.Z.)

**Keywords:** cognitive workload detection, air traffic controllers, mRMR, EEG channels

## Abstract

For air traffic controllers, the extent of their cognitive workload can significantly impact their cognitive function and response time, consequently influencing their operational efficiency or even resulting in safety incidents. In order to enhance the accuracy and efficiency in determining the cognitive workload of air traffic controllers, a cognitive workload detection method for air traffic controllers based on mRMR and fewer EEG channels was proposed in this study. First of all, a set of features related to gamma waves was initially proposed; subsequently, an EEG feature evaluation method based on the mRMR algorithm was employed to pinpoint the most relevant indicators for the detection of the cognitive workload. Consequently, a model for the detection of the cognitive workload of controllers was developed, and it was optimized by filtering out channel combinations that exhibited higher sensitivity to the workload using the mRMR algorithm. The results demonstrate that the enhanced model achieves the accuracy and stability required for practical applications. Notably, in this study, only three EEG channels were employed to achieve the highly precise detection of the cognitive workload of controllers. This approach markedly increases the practicality of employing EEG equipment for the detection of the cognitive workload and streamlines the detection process.

## 1. Introduction

In recent years, Chinese air traffic has become increasingly busy. The rise in traffic flow directly increases the workload of controllers, leading to a growing challenge in control. Consequently, accidents caused by controllers’ operational errors and human errors are gradually increasing. As is well known, individuals are susceptible to mental fatigue when involved in high-intensity cognitive tasks for extended periods. For air traffic controllers, the extent of their mental fatigue significantly impacts their cognitive function, response time, and vigilance, consequently influencing their operational efficiency. In severe instances, this fatigue may even result in safety incidents [1,2,3,4]. As a key group responsible for ensuring the safe and efficient operation of civil aviation, determining the controllers’ optimal cognitive workload is essential in guaranteeing the safety of aircraft operations in this sector. Thus, the accurate monitoring of their cognitive workload not only ensures optimal workload levels but also prevents controllers from being overloaded. This is an important task in the design of human–computer systems. Therefore, researching and monitoring controllers’ cognitive workload is of significant practical and applied importance.

### 1.1. Related Work about Cognitive Workload

The cognitive workload is typically defined as the amount of information processing capacity or cognitive resources needed to meet the current demands [5]. These cognitive resources mainly refer to the attentional resources and working memory capacity involved in cognitive processes [6]. Since the brain has limited cognitive resources, individuals have a restricted capacity and duration in which to perform cognitive tasks. Prolonged heavy tasks can lead to cognitive overload, which can impact task performance and increase the likelihood of errors [7]. Therefore, assessing the cognitive workload is crucial in enhancing individuals’ work and study efficiency and reducing error rates.

At present, cognitive workload detection methods are mainly divided into three categories: subjective detection methods, task measures, and physiological measures. Subjective testing methods include the Karolinska Sleepiness Scale, NASA-TLX scale, etc. [8,9]. Frequent filling of the scale, however, may disrupt the actual work of the experimental subjects, and it will also add to the workload. The task detection method involves adjusting the task difficulty to manipulate the level of task load and identifying changes in task difficulty through operational performance indicators [10]. However, performance measurement is task-based, and it is difficult to compare the results across tasks when different dimensions are used. For example, the task of controlling the allocation of attention involves both control and monitoring. While control is easy to measure, monitoring is difficult to measure. In many cases, most systems are not equipped to provide information on operator performance, making it difficult to obtain direct operational data on performance. Physiological detection is mainly divided into EEG signal detection, ECG signal detection [11], eye information detection [12], facial feature detection, and speech feature detection [13]. Objective detection methods rely on physiological data, which can accurately capture physiological indicators, exhibit high reliability and validity, and reflect the real-time status. Among the existing objective methods, ECG signals, eye and facial information, and EEG signals can reflect the real-time state of the controller and provide a basis for real-time assessment. Among these methods, EEG signals have the highest accuracy and have been verified to be reliable in assessing the cognitive workload of controllers [14,15]. EEG signals have become an important technique for the evaluation of cognitive workloads. Existing studies have shown that EEG signals are sensitive to the brain load of subjects for various tasks and the different difficulty levels of the same task. Therefore, they can be utilized as indicators to assess the cognitive load of subjects and determine the memory sources of learners [16,17].

Studies based on spontaneous EEG signals typically examine the variations in each frequency band at different cognitive workload levels. Algorithms like pattern recognition are utilized to construct a brain load identification model, which is the most commonly employed method in cognitive workload research. It is customary to classify spontaneous EEG into five different rhythms according to the frequency bands: delta (δ), theta (θ), alpha (α), beta (β), and gamma (γ) waves. It has been shown that the energy values of the theta, alpha, and beta frequency bands are sensitive to changes in cognitive workload. Different waveforms can reflect various mental states of the brain. Different brain waves reflect various mental states and can provide insights into the brain’s functioning. Delta waves are linked to deep relaxation and restful sleep, whereas theta waves are prominent during trance or hypnagogic states. Positioned between conscious thought (beta) and subconscious thought (theta), alpha waves exert a calming influence and facilitate deeper relaxation and contentment. Beta waves are the most prevalent high-frequency waves during wakefulness, while gamma waves indicate heightened cognitive activity, indicating increased neuronal activity [18].

In 2010, Dasari et al. [19] conducted a study where they continuously monitored the EEG changes of civil aviation controllers during 2 h of simulator tests. It was concluded that the theta, alpha, and beta waves could be used to analyze the shifts in the mental states of the controllers. The results of this study were endorsed by the FAA Aviation Administration. Shou et al. [20] analyzed EEG data and discovered that the theta waves fluctuated significantly with the controller’s workload and the increase in the peak value of time. This finding suggests that the theta waves could more accurately reflect the changes in the EEG features resulting from the controller’s increased workload. Budi Thomas Jap et al. [21] analyzed four EEG features using a Fast Fourier Transform: (theta + alpha)/beta, alpha/beta, (theta + alpha)/(alpha + beta), and theta/beta. They found that the proportion of alpha waves was relatively increased while that of beta waves decreased during severe brain fatigue. Additionally, the ratio of (theta + alpha)/beta showed a significant increase. Arico et al. [15] verified the reliability of brain load assessment over time by analyzing the frontal and occipital theta waves and parietal alpha waves of control trainees after a simulator test. Borghini et al. [22] analyzed the effect of the cognitive workload on brain activity using EEG signals and found that as the cognitive workload deepened, the theta wave energy increased while the alpha wave energy decreased.

The current research results show significant progress in the study of EEG signal deconstruction and the impact of the cognitive workload on human behavior. In terms of EEG frequencies, workload detection for controllers generally includes the characterization of four bands, the delta, theta, alpha, and beta waves, with less consideration given to the role of gamma waves in the analysis of mental states [23]. This is because, previously, high-frequency signals were considered exclusively as noise in the context of computer technology and were therefore systematically filtered out [24]. Currently, there is still a gap regarding the analysis of high-frequency brain activity for controllers, although technological advances have made high-frequency brain activity analysis increasingly more mainstream. High-frequency waves, which represent high-level cognitive activities [25], are more suitable for the analysis of controllers’ mental states.

### 1.2. Related Work about Indicator Evaluation Methods

In addition, when applying the EEG power spectrum indicator to analyze the cognitive workload of controllers, the applicability of different indicators varies and indicator evaluation methods have been developed. Currently, three types of indicator evaluation methods are widely utilized in research: those based on mutual information theory, statistics, and the separability of features.

Mutual information correlation methods typically select high-performing features by examining the degree of correlation between the features and the target variable. Shaibal Barua [26] evaluated three different feature selection algorithms and concluded that the BSS/WSS (BSS denotes “Between Sum of Squares” and WSS denotes “Within Sum of Squares”) method emphasizes discrepancies between different feature classes, whereas Sequential Floating Forward Selection (SFFS) prioritizes the internal relationships among features. The mRMR algorithm offers a balanced approach, yielding a more concise feature set than BSS/WSS while demonstrating superior generalization compared to SFFS. Arvaneh et al. [27] implemented indicator evaluation and ranking based on the mutual information algorithm, while Cheema et al. [28] extracted various EEG-based features and selected 15 using the mRMR algorithm.

Indicators can also be selected based on statistical principles, typically through the correlation coefficients of indicators in different conditions or by identifying significant differences between features. For instance, Li Wei [29] calculated 12 energy parameters and utilized gray relational analysis (GRA) to identify optimal features for the detection of driver fatigue using EEG data. Aghajani et al. [23] ranked indicators based on the correlation coefficients between the indicators and cognitive workload using the Pearson correlation coefficient, while Thiago, L.T. [30] employed the Wilcoxon signed-rank test to validate the performance of specific indicators in drowsiness detection. Additionally, Zhang, P. [31] utilized the Shapiro–Wilk (SW) test and ANOVA method to examine differences in EEG features across different brain fatigue states. Logistic regression was then used to identify features sensitive to the fatigue state based on their AUC and *p* values.

Some scholars have also assessed indicators through the separability of features, as demonstrated by Hu, J. [32], who utilized ten advanced classifiers to categorize the features, revealing that the choice of classifier significantly impacts the accuracy and the performance of indicators across different classifiers is not consistently aligned.

In the above approach, the feature selection algorithm based on mutual information theory considers the amount of mutual information and redundancy between features and selects a subset of features by iterating and removing features. However, existing EEG devices are generally multichannel, and such algorithms tend to focus more on calculating the redundancy between features while ignoring the redundancy of features across channels. The evaluation of feature performance through the Wilcoxon signed-rank test only determines whether features exhibit significant changes, lacking the ability for comparative and quantitative assessment. Meanwhile, gray relational analysis (GRA) offers the comprehensive consideration of multiple factors and displays a degree of robustness against incomplete, imprecise, or noisy data. Nevertheless, GRA requires data normalization, increasing its computational complexity. Additionally, the subjective nature of determining weighting parameters and the calculation method of the gray correlation in GRA may introduce subjectivity, potentially affecting the final outcomes.

Furthermore, the classifier-based indicator evaluation approach is heavily influenced by the choice of classifier and classification method, resulting in different behaviors of the same feature across various classification methods. This variation in feature evaluation is compounded by the parameter settings within the same classifier, contributing to challenges in achieving the uniform assessment of features.

In conclusion, the indicator with the highest precision in detecting controllers’ cognitive workload still needs further analysis [33].

### 1.3. Related Work about EEG-Based Methods with Few Channels

Further, there are still some issues that need to be addressed when applying EEG equipment to detect the cognitive workload in controllers: the portability of the equipment and the complexity of the detection operation. It is important to note that a multichannel EEG acquisition system, such as the 64-channel EEG system used in the experiments in this study, is a relatively complex piece of equipment. Trained technicians are required to position the electrodes, as all electrodes must be placed in the appropriate locations. In addition, applying EEG paste to each electrode individually is also very time-consuming. All of these factors make it difficult to apply the system in real work scenarios. Therefore, an EEG system suitable for practical use must be a portable system with fewer channels to estimate the controller’s workload. This system should not only be cost-effective but also simple to operate and user-friendly.

Based on the complex nature of EEG signals with multiple channels and its practical implications, various channel selection strategies have been developed. Tavakolian et al. [34] introduced a channel reduction method using a subset generation genetic algorithm for the classification of mental tasks, employing a random search approach to select a combination of six optimal channels from a pool of 19 channels. Lan et al. [35] employed a sequential search method with filtering techniques for subset channel selection. With 32 EEG recording channels, the average classification accuracy for all subjects reached around 80% when using seven, ten, and seven channels. Alyasser et al. [36] proposed an EEG-based binary flower pollination algorithm (FPA) and a β-climbing method for individual identification channel selection, demonstrating that reducing the number of channels can significantly improve the accuracy rates.

In conclusion, it has been demonstrated that fewer channels of EEG signals can be used to detect alertness levels and fatigue [26,37,38], but their use for workload detection still needs to be verified. From a practical point of view, research should identify the most relevant EEG channels, which would reduce the complexity of the sensor setup and maintain its performance [39]. In this study, our purpose is to optimize the detection process by employing the minimum number of channels necessary. To achieve this, the selected channels must contain significant mental workload-related information with minimal redundancy. However, the above techniques are not fully applicable to this objective; therefore, we introduce an algorithm based on mutual information theory to complete the channel selection work. This algorithm uses mRMR and it allows for a reduction in the channel count while preserving crucial information, streamlining the detection task with a limited channel selection.

**Figure 1:**
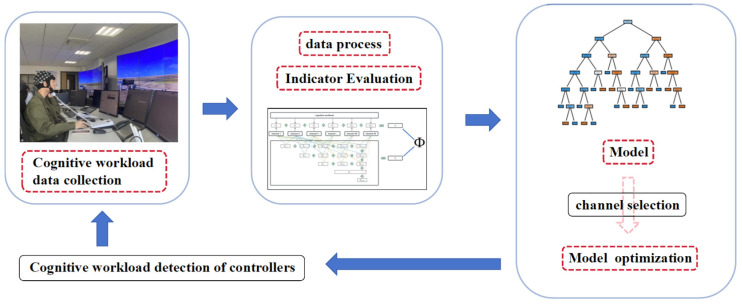
Framework diagram.

To address the aforementioned challenges, this study initially designed an experiment to obtain cognitive workload data from controllers; then, it delineated a series of power spectrum features associated with gamma waves and developed an efficient method for the evaluation of these indicators to discern the best-performing ones. Subsequently, to augment the accuracy and efficiency of cognitive workload detection, a workload detection method for controllers was developed and optimized from the perspective of channel reduction, making it more suitable for practical applications. The workflow of this study is shown in Figure 1.

The novelty of this work is evident in three main aspects.

(1) Introducing EEG features linked to gamma waves and evaluating their applicability through comparisons with conventional features in controller cognitive workload detection.
(2) Proposing an EEG feature evaluation method based on the mRMR algorithm. This technique circumvents the subjectivity inherent in the gray correlation analysis method when determining weight parameters and the gray correlation degree, as well as the variability associated with classifier evaluation methods. Compared to existing feature selection algorithms, this approach considers information redundancy within the channel, resulting in more accurate evaluation outcomes. Additionally, unlike statistically based correlation techniques, this method enables the quantitative evaluation of the feature performance, enabling meaningful comparisons across different features.
(3) Developing a cognitive workload detection method based on three channels for controllers, successfully achieving cognitive workload detection for 41 subjects using only three channels.

## 2. Materials and Methods

### 2.1. Experiments and Data

#### 2.1.1. Subjects

In research, having fewer subjects may result in model overfitting and poor generalization. In integrated models, generalized patterns and common features are typically extracted from the data of multiple individuals. This means that a larger number of subjects is required. For these reasons, we sent invitations to local controllers and ultimately invited 41 controllers from Nanjing Airport and the Jiangsu Air Traffic Control Bureau, who held valid control licenses, to participate in the experiment.

In order to minimize the inter-subject differences caused by gender, we invited male subjects aged between 26 and 34 years old. All subjects provided informed consent before participating in the study. They were trained to proficiently operate the control simulation system before the official experiment began. For example, they needed to understand how to use the simulator system to issue commands, how to use the system’s call features, and so on. The formal experiment could only begin after ensuring that all subjects could skillfully use the simulator to accomplish the controller’s work tasks.

In addition, each participant had not worked night shifts before the experiment and was free from prescription drug use and medical contraindications, such as serious concomitant illnesses, alcoholism, substance abuse, and psychological or intellectual issues that could affect their compliance.

#### 2.1.2. Apparatus and Experimental Site

The EEG signal acquisition equipment included the Borecon NeuSen W series wireless EEG acquisition system (64 channels), a portable computer for the receiving and collection of EEG signals, conductive paste, and syringes.

Control-related equipment: Since the traffic flow cannot be controlled in the actual control scenario, we chose to conduct the experiment on a control simulator. The control simulator was provided by Nanjing University of Aeronautics and Astronautics.

Experimental Site: Due to the extremely weak nature of the brain signals on the scalp, they are highly susceptible to interference from the power frequency, the internal noise of electronic instruments, and magnetic fields during signal acquisition. Considering the requirements of the experimental equipment, it is preferable to conduct experiments in shielded electromagnetic rooms. Therefore, the experiment was conducted in a quiet, enclosed, and empty simulator laboratory.

#### 2.1.3. Scenario Design

Generally speaking, the traffic flow is one of the main factors that affects the workload of air traffic controllers. It refers to the number of aircraft that need to land or take off within a specific period. This is typically measured by counting the number of landings and takeoffs. When the air traffic increases, air traffic controllers need to handle more aircraft simultaneously and ensure that they operate safely and efficiently in the air and on the ground [40]. A higher traffic flow necessitates the assignment of more aircraft to suitable routes, altitudes, and intervals. This task requires air traffic controllers to maintain constant vigilance over multiple aircraft operating simultaneously and to provide timely and accurate decisions and instructions. Therefore, an increase in the traffic flow will increase the workload and pressure on air traffic controllers.

However, in addition to the traffic flow, other abnormal situations may also affect the workloads of air traffic controllers, such as equipment failures, foreign objects on runways, the special operational requirements of airports, and incident handling. In abnormal situations, air traffic controllers need to process information, make decisions, and provide guidance based on various scenarios to ensure aviation safety. By questioning the controllers, we learned that, in the case of an abnormal situation, controllers need to consider measures such as resuming flights, avoidance, emergency evacuation, and standby to ensure the safe and efficient operation of air traffic. Dealing with such a situation will increase their cognitive workload and also lead to greater psychological pressure on them.

**Figure 2:**
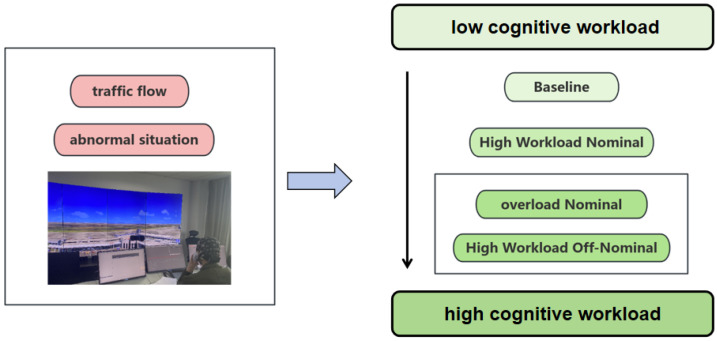
Factors Influencing cognitive workload of air traffic controllers.

As shown in Figure 2, for the reasons mentioned above, the workload of controllers is typically divided into three phases: baseline, high-workload nominal, and high-workload off-nominal [40]. The experimental scenario settings in this study aligned with the controller’s workload phases, as illustrated in Table 1. It was expected that the cognitive workload could be detected by analyzing the EEG data of the controllers in various work scenarios.

In this study, scenario 1 (the baseline phase) includes a maximum of 6 flights within a specified time period, representing a moderate workload, with no abnormal situations occurring during this scenario. Scenario 2 (the high-workload nominal phase) consists of a maximum of 21 flights, and, once again, there are no abnormal situations in this scenario. In addition to the three phases mentioned above, this study also introduces scenario 3 (the overload nominal phase) to provide a more comprehensive evaluation of the controller’s cognitive workload. This scenario involves managing 30 aircraft within a specified time frame without any abnormal situations. Scenario 4 (high-workload off-nominal) refers to an experimental setup that includes abnormal situations in addition to high-workload nominal. Here, the abnormal situation being discussed is the presence of a foreign object on the runway, a scenario that is frequently encountered in the daily tasks of controllers. In practice, when an air traffic controller discovers a foreign object on the runway, they need to take the following measures immediately: (1) notify the control tower promptly; (2) issue warnings and instructions to aircraft taking off and landing, informing the pilots of the foreign object on the runway and requesting that they take responsive measures as soon as possible—instructions may include a request for the aircraft to stop taking off or landing or to follow special procedures; (3) work closely with the ground operation personnel to determine the nature of the foreign object and the extent of the hazard as soon as possible and then coordinate closely with the operation personnel to ensure the prompt dispatch of personnel to the runway for inspection and clean-up efforts; (4) ensure that the progress of the foreign object clearance work and the safety situation is reported to the control tower and aircraft.

Before conducting the experiments, we designed pre-experiments to determine the sequencing of the scenarios. During the pre-experiments, the EEG data were classified by an SVM classifier under different scenario orders, such as “scenario 1—scenario 2—scenario 3—scenario 4” and “scenario 4—scenario 3—scenario 2—scenario 1”. According to the results, the EEG data of the subjects were most separable in the order of “scenario 4—scenario 3—scenario 1—scenario 2”, so the subsequent experiments were conducted in this order.

#### 2.1.4. Experimental Procedure

The experimental procedure is shown in the figure below.

**Figure 3:**
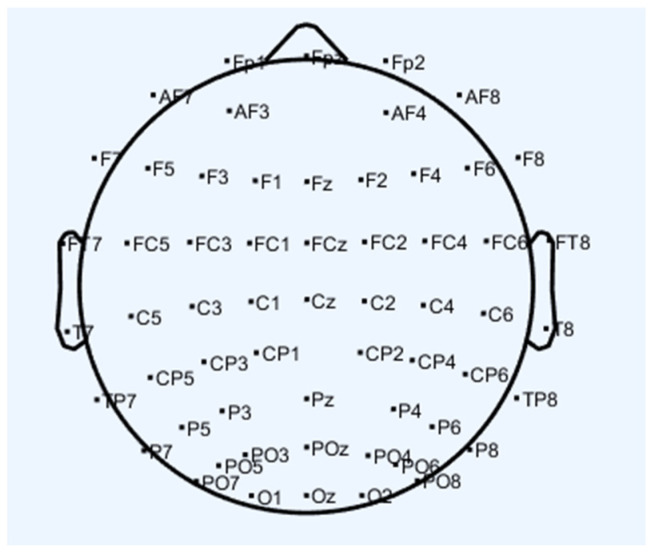
Electrode locations.

**Figure 4:**
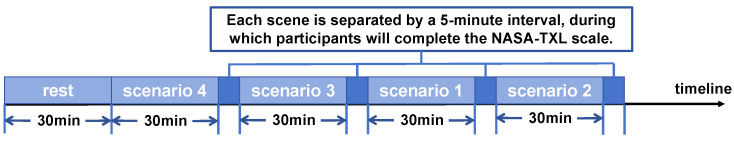
Experimental procedure.

(1) The subjects were asked to fill in their personal information, including their names and ages. Then, they were provided with an explanation of the experimental process to help them to understand the aspects related to the experiment and to minimize behaviors like swallowing, blinking, and shaking their legs.
(2) The experimenters placed the EEG caps on the subjects, installed the equipment electrodes (the electrodes were placed as shown in Figure 3), injected the EEG paste, and adjusted the relative positions of each part to ensure the comfort of the participants.

(3) The EEG data in the resting state were measured first to ensure that the equipment could be used normally, and then the simulator experiments for the four scenarios were initiated. After the completion of each scenario, the controllers completed the NASA-TXL workload scale for that particular scenario. We continuously recorded the EEG data of the controllers throughout the experiment. The specific process is illustrated in the Figure 4.

(4) At the end of the experiment, we stopped recording the controllers’ EEG data and saved the data.

### 2.2. Data Processing

#### 2.2.1. NASA-TXL Preprocessing

**Figure 5:**
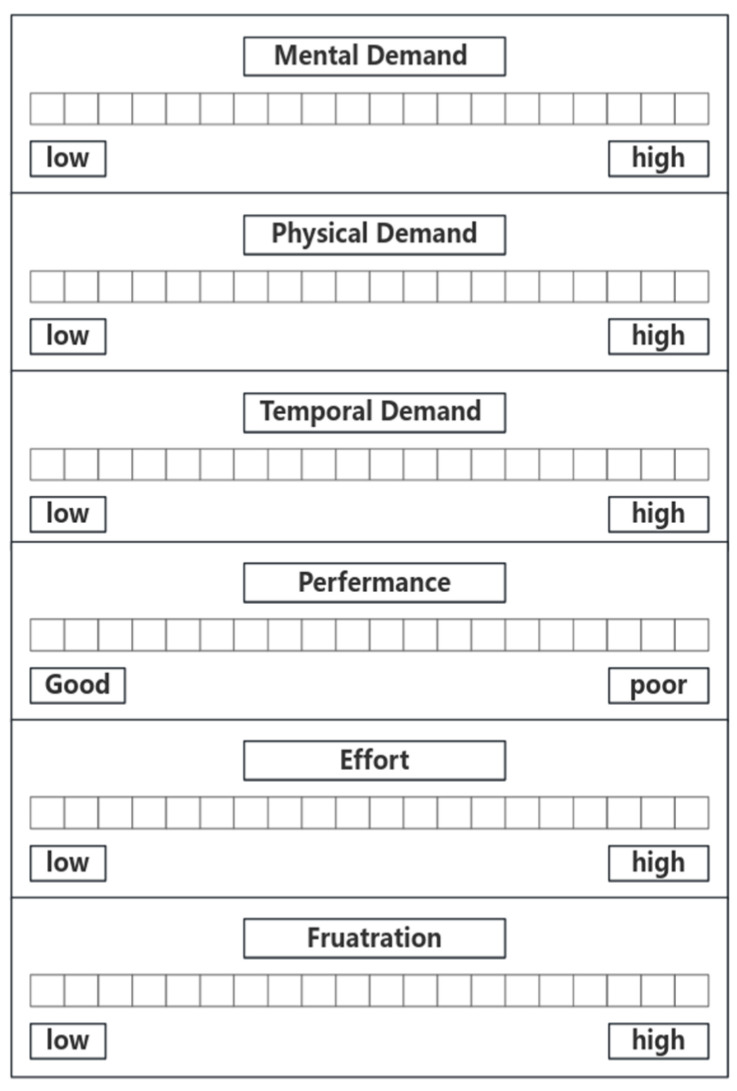
NASA-TXL scale used in the experiment.

The NASA Task Load Index (NASA-TLX) is a multi-dimensional assessment of workloads developed by NASA’s Ames Research Center. It is assessed on six scales: mental demands, physical demands, temporal demands, own performance, effort, and frustration, as shown in Figure 5.

**Figure 6:**
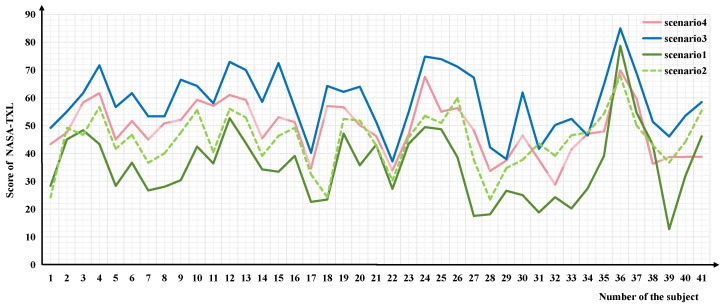
NASA-TXL data of 41 subjects.

As shown in Figure 6, we utilized the NASA-TXL to evaluate the cognitive workload [32] for each scenario using a 21-point scale, and the scores obtained were employed to characterize the cognitive workload. Data from the NASA-TXL scale were collected from 41 subjects to assess the four scenarios: baseline, high-workload nominal, overload nominal, and high-workload off-nominal. The results are shown in the table below. The subjects’ perceptions of their cognitive workload for each scenario varied slightly, but the overall trend was consistent: scenario 3 (overload nominal) had the highest load, while scenario 1 (baseline) had the lowest load. Scenario 4 fell between scenario 2 and 3 in terms of cognitive workload.

#### 2.2.2. EEG Data Processing

Before analyzing the EEG data, this study first carried out several preprocessing steps on the collected EEG data, such as eliminating unnecessary electrodes, re-referencing, filtering, performing ICA, removing artifacts, and so on.

### 2.3. Indicator Evaluation Method Based on mRMR Algorithm

#### 2.3.1. Feature Extraction

**Figure 7:**
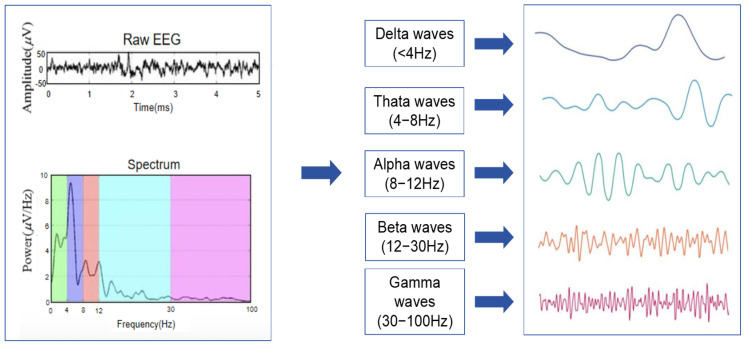
Frequency domain analysis.

Before evaluating the cognitive workload of controllers, it is necessary to select indicators that can be used to quantitatively characterize the workload. This study opts for frequency domain analysis to examine the EEG data of the controller. Frequency domain analysis involves converting EEG signals from the time domain to the frequency domain, as shown in Figure 7. The outcome of this analysis is the distribution of the energy values at each frequency, commonly known as the power value, which can be simplified as the square of the amplitude.

Traditional controller workload detection generally includes indicators of four bands: the δ wave, α wave, β wave, and θ wave. However, less consideration is given to the role of the γ wave in the analysis of mental states. In order to verify whether gamma-related indicators can be used to detect the cognitive workload, this study first considers the common traditional indicators by referring to existing indicators in the literature: α absolute energy, β absolute energy, δ absolute energy, θ absolute energy, α/(δ + β + θ + α), β/(δ + β + θ + α), θ/(δ + β + θ + α), (α + θ)/β, θ/β, α/β, and (α + θ)/(α + β) [21,41]. Building upon this foundation, indicators related to the γ wave, γ absolute energy, γ/(δ + β + θ + α), and γ/(δ + β + θ + α + γ) are proposed. These indicators are shown in Table 2. Indicators 1–13 are common working condition detection indicators, while Indicators 14–26 are new indicators for cognitive workload analysis related to the γ wave that are proposed in this work.

Although delta waves are usually associated with deep sleep, their manifestations and roles may vary in certain specific waking states or under specific physiological and pathological conditions. Our study aimed to comprehensively explore the potential effects of various brain waves in specific contexts and was not limited to traditional cognition. Therefore, delta waves and their associated metrics were also considered in this study.

#### 2.3.2. Evaluation Method of Indicators Based on mRMR Algorithm

**Figure 8:**
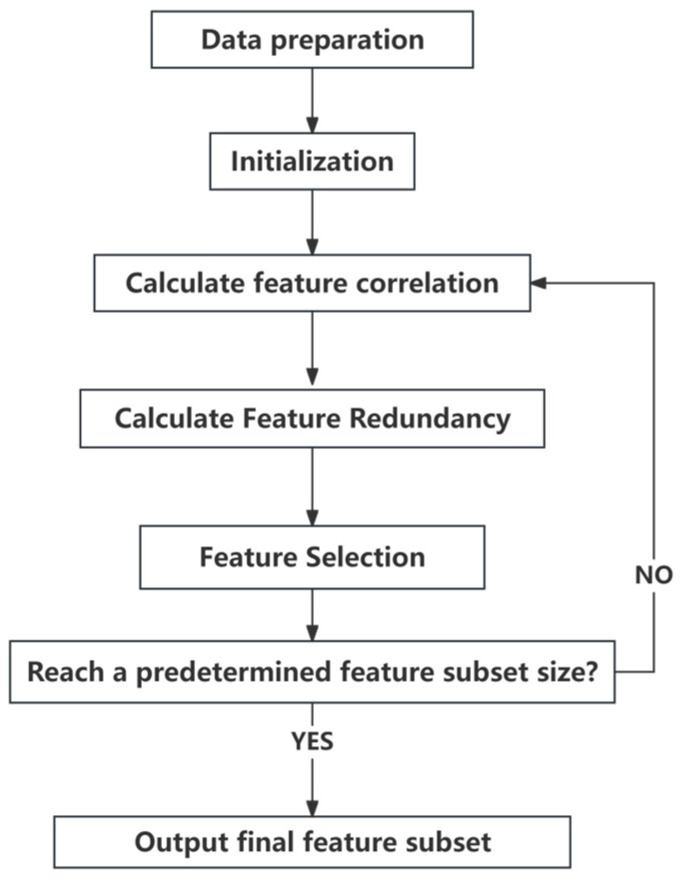
Flowchart of mRMR algorithm. In the data preparation phase, feature datasets and their corresponding target variables were collected. Then, during the initialization step, the empty initial feature subset was established. When calculating feature correlations, the relationship between each feature and the target variable was analyzed. Then, in evaluating feature redundancy, the degree of redundancy among features was assessed. Throughout the feature selection process, the optimal features to be added to the feature subset were selected. This iterative process involves continuously calculating the feature correlations, assessing the feature redundancy, and refining the feature selection until reaching a predetermined subset size, culminating in the output of the final refined feature subset.

The mRMR algorithm is typically used to select important features by maximizing the correlation between the features and the target variable while minimizing the redundancy between features. When selecting a subset of features, the mRMR algorithm iteratively adds and removes features to ensure that the chosen subset has both high relevance and low redundancy [42,43]. The method is based on information-theoretic principles and evaluates the importance of features by calculating the correlation and redundancy between them. It then selects the most representative subset of features. The specific steps of the algorithm’s implementation are as Figure 8.

When evaluating the correlation between the power spectrum indicators and cognitive workload, two issues need to be addressed: the extent to which the indicator values in each channel correlate with the cognitive workload and the level of redundancy present in the information across different channels. When these two issues are resolved, we can find an indicator that correlates strongly with the cognitive workload and has minimal redundancy across channels. These indicators will help to filter out combinations of channels that are independent of each other. In this way, the detection of cognitive workloads with fewer channels can be realized.

Therefore, we need to consider how to measure the relevance between the indicator and cognitive workload, as well as how to reduce the redundancy between the channels. To address these two issues, this study introduces the mRMR algorithm to evaluate the indicators. Since the mRMR algorithm is primarily used for feature selection rather than evaluating the indicators that we need, innovations in the application of the mRMR algorithm have been made for work evaluation purposes.

Since the focus here is on the comparison of the indicators, a quantitative metric is needed to evaluate the performance of the power spectrum indicators proposed above. In this study, “metric” Φ is defined as a measure that evaluates the correlation between the power spectrum indicator and cognitive workload. The calculation methodology is presented below.

Let $x_{i}$ denote a channel i and S denote the function of channels for an indicator. In the mRMR algorithm, c is usually defined as the target variable, and, in this study, c denotes the cognitive workload of controllers. After eliminating 5 useless channels, 59 channels remain, so, in this study, |S| = 59. Considering all 59 channels collectively, the mutual information between the 59 channels and the workload is initially calculated to satisfy the following function:

(1) $$\max D(S,c),D=\frac{1}{S}\sum_{x_{i}\in S}I(x_{i};c)$$

However, the features selected through Max-Relevance may exhibit redundancy across channels. When two features are redundant, removing one of them does not significantly alter the classification result. Therefore, the Min-Redundancy approach can be utilized to eliminate redundant features. Subsequently, the sum of redundancy R(S) within the 59 channels needs to be calculated, as shown in the following equation:

(2) $$\min R(S)=\frac{1}{{\left|S\right|}^{2}}\sum_{x_{i},x_{j}\in S}I(x_{i},x_{j})$$

The ultimate goal is to determine the maximum correlation value and minimum redundancy of all of the indicators and then rank them to assess the performance of the power spectrum indicators suggested earlier. The objective function is as follows:

(3) maxϕ(D,R),ϕ=D−R

**Figure 9:**
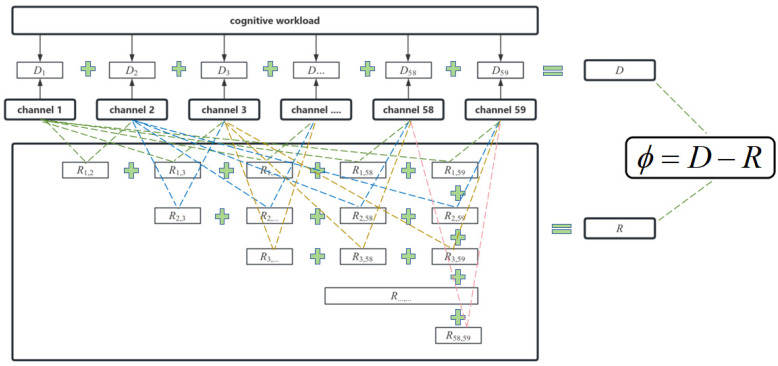
The calculation process of the metric is illustrated in the figure. Each channel represents the amount of information that it contains for a specified feature. By calculating the mutual information corresponding to the indicator between each channel and the cognitive workload, as well as the redundant information between channels, the value of ϕ can be obtained and an evaluation can be performed on the indicator.

In order to illustrate the calculation process more intuitively, it is depicted in Figure 9.

### 2.4. Cognitive Workload Detection Method Based on Fewer Channels

#### 2.4.1. Workload Detection Model

Through the above method, the best-performing indicators can be selected. Further, based on these indicators, the workload of the controllers can be detected. Generally, cognitive workload detection or fatigue detection couples the indicator set as input. However, the calculation of the indicator set will undoubtedly slow down the speed of the calculation and cause a certain degree of interference in practical applications. In addition, the indicator set generally consists of multiple indicators. The optimal detection channel or combination corresponding to different indicators might vary widely, making it difficult to narrow down the channels for the channel set. For these reasons, we chose to input only the data of the best-performing indicator to establish a decision tree model to detect the cognitive workload of controllers.

When constructing the model, leave-one-out cross-validation (LOOCV) was employed to prevent overfitting [44], where each subject’s sample served as the validation set in a rotating fashion. This approach ensured that each subject’s sample was validated and provided a more precise evaluation of the model’s performance. Moreover, the maximum depth of the tree was set to 10. The accuracy, recall, precision, and F1 score were calculated as the performance metrics to evaluate the performance of the model [45]. Among them, accuracy measures the proportion of correctly classified samples out of the total samples. Precision is the ratio of correctly predicted positive samples to all predicted positive samples. Recall assesses the proportion of correctly predicted positive samples out of all true positive samples. The F1 score is a composite metric that considers both recall and precision, calculated as the harmonic mean of the two [46]. To enhance the model’s accuracy, we employed the grid search method to identify the optimal parameter combinations. Grid search is a method that exhaustively tests all parameter combinations within a given range to find the parameter settings that perform best in the model. Grid search is a common method used for parameter tuning in the field of machine learning and has been widely demonstrated to be robust [47].

#### 2.4.2. Optimization and Validation of Workload Detection Model

Although the model above can be used for detection, locating the electrodes of an EEG device and applying EEG paste to multiple electrodes one by one is still inconvenient for practical applications [32]. For this reason, we need to consider filtering the channels, eliminating inefficient channels, and retaining channels or channel combinations with higher accuracy. The question of how to reduce the number of channels while maintaining the accuracy is addressed in this paper.

As the mRMR algorithm is a feature selection algorithm that iteratively adds and removes features, resulting in a subset of selected features with a strong correlation and low redundancy, this algorithm can be directly used to identify channels that are more sensitive to the workload. The set of channels selected by this algorithm has a strong correlation with the cognitive workload. Meanwhile, different channels in the set have low redundancy.

After model optimization, the generalizability of the model will be further validated. Research has demonstrated that the robustness, accuracy, and reliability of cognitive workload detection based on physiological signals require further enhancement when applied in real operating conditions [48]. Hence, to enhance the model’s applicability, this study conducted validation experiments to acquire EEG data from diverse subjects and employed a novel dataset to validate the model’s generalizability. In the aforementioned experiment, to minimize inter-subject variability, all subjects were male. To verify the usability of the model in subjects of different genders, this study invited four licensed controllers to complete the experiment, among whom were two male controllers and two female controllers. After data collection, the optimal features of the best channel combinations were extracted and input into the model, and the generalizability of the model was validated based on the output results.

Based on the above methods, we expected to be able to detect the cognitive workload of controllers in an efficient and convenient way.

## 3. Results

### 3.1. Indicator Evaluation Results

After feature extraction, the indicator evaluation method based on the mRMR algorithm mentioned earlier was utilized to compute the value of each indicator. The calculation results are shown in the table below.

From Table 3 it can be observed that indicator 16 β + θ + α + γ, indicator 15 δ + β + θ + α + γ, indicator 5 δ + β + θ + α, indicator 14 γ, and indicator 4 β exhibit better performance compared to all other indicators. This suggests that these five indicators have a strong correlation with the cognitive workload and can be utilized to detect the workload of controllers. The classification outcomes in this study align with existing research [49], highlighting the efficacy of utilizing combined frequency rhythms over individual rhythms for classification purposes. Moreover, the EEG features related to gamma waves introduced in this study exhibit superior suitability for the detection of the mental workload in controllers compared to conventional features.

Meanwhile, indicator 6 δ/(δ + β + θ + α) and indicator 26 γ/(δ + β + θ + α + γ) showed weaker correlations with the workload. Indicators 7 θ/(δ + β + θ + α), 8 α/(δ + β + θ + α), 9 β/(δ + β + θ + α), 17 γ/δ, and 25 γ/(δ + β + θ + α) did not show a correlation with the workload.

Among the indicators in the five bands, γ and β outperformed δ, θ, and α, which was generally consistent with existing findings [50]. In other words, the signals linked to gamma and beta waves are more relevant for the detection of workloads.

**Figure 10:**
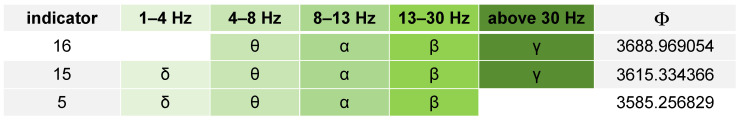
Comparison of the top 3 indicators.

Another result in Figure 10 is consistent with this conclusion: the result of indicator 16 β + θ + α + γ is better than that of indicator 15 δ + β + θ + α + γ and better than that of indicator 5 δ + β + θ + α. Comparing indicator 5 δ + β + θ + α with indicator 15 δ + β + θ + α + γ, it can be seen that the absolute energy of indicator 15 δ + β + θ + α + γ shows a stronger correlation with the cognitive workload after the addition of gamma. This suggests that the total energy containing the gamma wave is considered better in reflecting the changes in cognitive workload. From this, we can also draw the conclusion that the effects brought by γ waves can be considered greater when studying the controller’s cognitive workload.

Moreover, comparing indicator 16 β + θ + α + γ with indicator 15 δ + β + θ + α + γ, it can be observed that the addition of δ causes indicator 15 δ + β + θ + α + γ to perform more poorly; this was also in line with a previous study [51]. In conclusion, different indicators are discussed in this section, but not all of them are useful. Moreover, from the above, it can be concluded that the beta and gamma signals are appropriate to analyze the problem posed in this study.

### 3.2. Validation of Evaluation Results

In order to validate the conclusions drawn above, the SVM classifier [52] was used to classify the indicator data using various classification methods: resting/working, 0/low–medium/high load, 0/low/medium–high load, and 0/low/medium/high load. From this, an evaluation of the indicators could be achieved based on their separability. The classification results are shown in Table 4.

From the table below, it is evident that dividing the controllers’ EEG data into two categories, a resting state and working state, results in all indicators having accuracy above 0.8. The binary classification results suggest that the data can be effectively segmented, and the indicators are deemed valid. Among them, indicator 16 has the highest classification accuracy of 0.86, followed by indicators 5 and 15 at 0.84.

When classifying controllers’ workload into three categories, 0 load, medium–high load, and low load, the classification accuracy of all indicators can reach 0.6 and above, indicating that the data can be classified. Among them, indicator 16 β + θ + α + γ, indicator 15 δ + β + θ + α + γ, indicator 5 δ + β + θ + α, indicator 14 γ, and indicator 4 β perform better, with classification accuracy of 0.74, 0.66, 0.62, 0.62, and 0.62, indicating that these six indicators have a strong correlation with the workload, and the rankings match the above indicator evaluation rankings.

When the controllers’ load is classified into four categories, 0 load, low load, medium load, and high load, the classification accuracy of all indicators is much higher than the accuracy of random classification, at 0.25. Among them, indicator 16 β + θ + α + γ, indicator 15 δ + β + θ + α + γ, indicator 5 δ + β + θ + α, and indicator 14 γ, as well as indicator 4 β, perform better. This suggests that these six indicators are more relevant to the workload, and their rankings align closely with the aforementioned indicator evaluation rankings.

In conclusion, indicator 16 β + θ + α + γ has the highest accuracy, regardless of the type of classification. Indicator 15 δ + β + θ + α + γ, although slightly less accurate than indicator 16, is ranked second in accuracy across all classification methods, followed by indicators 5 δ + β + θ + α, 14 γ, and 4 β. Meanwhile, indicators 6, 7, 8, 9, 16, 25, and 26 are less separable and ranked lower.

**Table 4:** Classification accuracy.

Indicator	Resting/ Working	0/Medium–Low /High Load	0/Low/ Medium–High Load	0/Low/Medium /High Load
Indicator 1	0.82	0.44	0.6	0.42
Indicator 2	0.82	0.47	0.6	0.42
Indicator 3	0.82	0.47	0.61	0.44
Indicator 4	0.82	0.5	0.62	0.43
Indicator 5	0.84	0.55	0.62	0.49
Indicator 6	0.82	0.38	0.6	0.43
Indicator 7	0.82	0.37	0.6	0.43
Indicator 8	0.82	0.37	0.6	0.43
Indicator 9	0.82	0.37	0.6	0.43
Indicator 10	0.82	0.43	0.6	0.41
Indicator 11	0.82	0.4	0.6	0.43
Indicator 12	0.81	0.42	0.6	0.41
Indicator 13	0.82	0.42	0.6	0.43
Indicator 14	0.82	0.47	0.62	0.46
Indicator 15	0.84	0.57	0.66	0.5
Indicator 16	0.82	0.38	0.6	0.43
Indicator 17	0.82	0.39	0.6	0.43
Indicator 18	0.82	0.41	0.6	0.44
Indicator 19	0.82	0.45	0.6	0.43
Indicator 20	0.82	0.37	0.6	0.43
Indicator 21	0.82	0.38	0.6	0.43
Indicator 22	0.82	0.42	0.6	0.43
Indicator 23	0.82	0.38	0.6	0.43
Indicator 24	0.82	0.37	0.6	0.43
Indicator 25	0.82	0.37	0.6	0.43
Indicator 26	0.86	0.64	0.74	0.55

As can be seen from the table above, under the classification categories of resting/working and 0/low/medium/high load, the difference in accuracy between the features is small, and there are even many features with the same classification accuracy, so it is difficult to select features for the subsequent analysis.

Under the two classification divisions of 0/low/medium/high load and 0/low/medium/high load, there is a larger difference in the accuracy, but the ranking of the features under these two classification methods also shows a difference. Some features have higher accuracy under the classification of 0/medium-low/high load, while some features have higher accuracy under the classification of 0/low/medium/high load, which makes it difficult to select features for the subsequent analysis.

To enable comparisons between indicators, the data were normalized using the following formula:

(4) $$f(x_{i})=\frac{x_{i}-x_{\min}}{x_{\max}-x_{\min}}$$

$f(x_{i})$ is defined as the value of the indicator after normalization, and it provides a value between 0 and 1. A value of 0 indicates the lowest accuracy under the corresponding classification method and 1 indicates the highest accuracy under the classification method. The normalization results are presented in the following table.

**Figure 11:**
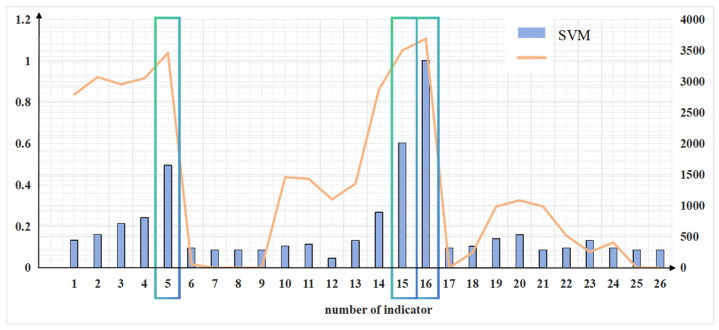
Comparison of the two methods.

After normalization, the mean value $f(x_{i})$ of each indicator under the four classifications was obtained, and it is depicted in Figure 11. The better-performing indicators (for the green square) identified by the two approaches are consistent and include β + θ + α + γ (indicator 16), δ + β + θ + α + γ (indicator 15), and δ + β + θ + α (indicator 5).

Overall, the rankings of the indicators obtained by the mRMR algorithm are essentially consistent with the SVM ranking results. Therefore, we can conclude that the proposed evaluation method is effective. Compared to the evaluation method based on the mRMR algorithm, using a classifier for feature selection has certain flaws. For example, in the classification method of rest/working and 0/low/medium–high workload, the differences in classification accuracy between the indicators are minimal. In fact, many indicators have the same classification accuracy, making it difficult to choose indicators for the subsequent analysis. In the classifications of 0/low/high workload and 0/low/medium/high workload, the indicators show significant differences in classification accuracy. However, the ranking of the indicators varies greatly between these two classification methods. Some indicators perform better in the 0/low/high workload classification, while others excel in the 0/low/medium/high workload classification, thereby increasing the complexity of feature selection.

### 3.3. Results of Workload Detection Model

**Figure 12:**
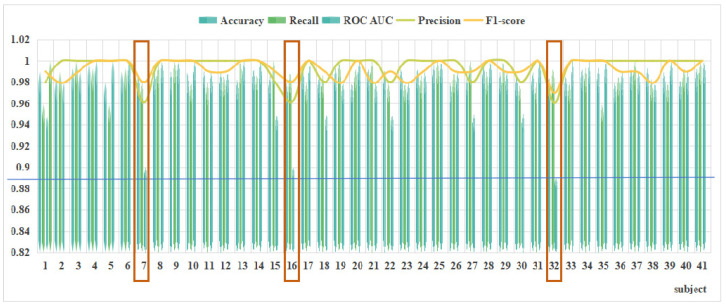
Results of the model based on the full channel.

The detection accuracy and precision of different subjects in the decision tree model were obtained, as shown in Figure 12.

First, accuracy is paramount. For the 41 subjects, the model’s accuracy ranges from 0.996 to 0.979. In other words, the model’s accuracy fluctuates within 0.9875 ± 0.0085. This result shows that the model’s accuracy meets the requirements for application and it can be utilized for workload detection in controllers.

Next, precision is considered. The value of precision fluctuates between 0.96 and 1. For the vast majority of subjects, the precision can reach 1, indicating that the model is highly accurate. In addition to this, both the recall and F1 score values are as expected, with recall values of 0.9795 ± 0.0205 and F1 score values as high as 0.969.

**Figure 13:**
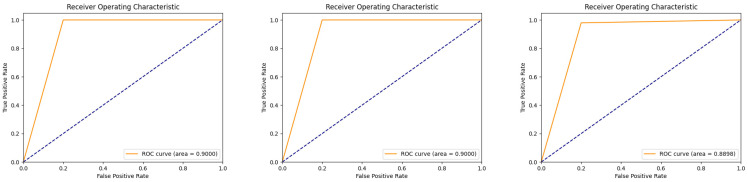
ROC AUC of subjects 7, 16, and 32.

However, the ROC AUC of the model was not as promising as the aforementioned metrics, with the exception of subjects 7, 17, and 32, where the ROC AUC values could reach up to 0.95 to 1, suggesting that this metric performed better in most of the subjects. However, the metrics for subjects 7, 16, and 32 were poor, ranging from 0.889 to 0.90, as shown in Figure 13. This indicates that the predictive performance of the model is relatively poor for these subjects.

For most of our subjects, the workload model performed as expected and could be used for their workload detection. However, as shown in the figure above, the performance of the metrics was relatively poor for subjects 7, 17, and 32. This suggests that the workload detection model is not optimal for these three subjects.

Nevertheless, in practical applications, the detection accuracy for these subjects can already meet the needs, and the model is suitable for most subjects. Although it may not be optimal for a few subjects, the detection accuracy meets the usage criteria, indicating that our model can be applied in practical work scenarios.

### 3.4. Results of Optimization

**Figure 14:**
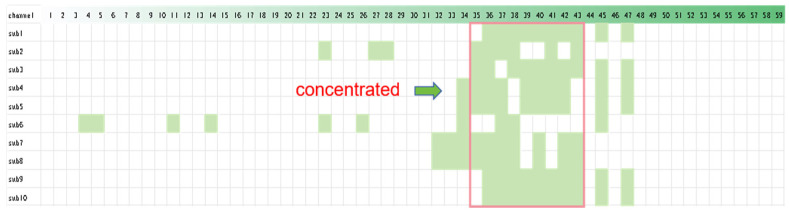
Results of channels selected by mRMR.

Although the model above can already meet the needs in terms of detection accuracy, locating the electrodes of an EEG device and applying EEG paste to multiple electrodes one by one is still inconvenient in practical applications. The channel selection results obtained using the mRMR algorithm are depicted in Figure 14, which displays the top 10 channels for the 10 subjects. Contrary to our expectations, the optimal channel combination for each subject is not consistent. This suggests that there is variability among individuals, and the optimal channel combination varies from person to person. Therefore, we cannot choose a channel combination that is optimal for everyone.

However, even though the optimal channel combination varies for each subject, we can observe that the superior channels are concentrated in their distribution. From the figure, we can see that the optimal channel combination for each subject occurs with one of the channels 35, 36, 37, 38, 39, 40, 41, 42, or 43. Although they do not appear simultaneously, their combination will ensure that one of the best channel combinations for all subjects is always achieved. This suggests that we can identify a channel combination that, although not optimal for everyone, achieves detection accuracy that is as high as possible across all subjects.

**Figure 15:**
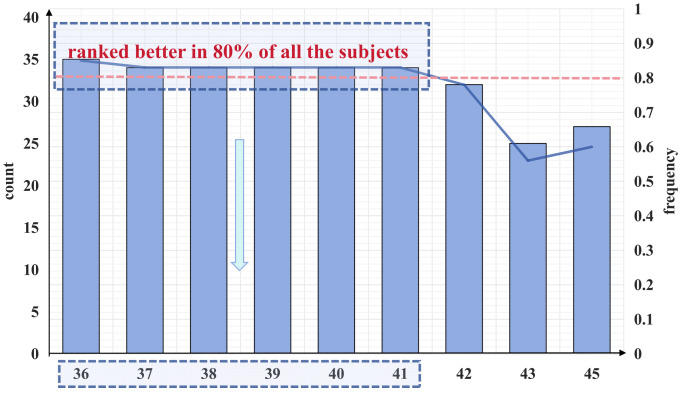
Statistical map of channels with a high frequency of occurrence.

Accordingly, we analyzed the results of the channels using the mRMR algorithm for 41 subjects. As shown in Figure 15, channels 36 (CP2), 37 (CP3), 38 (CP4), 39 (CP5), 40 (CP6), and 41 (TP7) appear more frequently, exceeding 80%. This indicates that these channels are ranked higher in 80% of the subjects. When evaluating the workload of the controllers, we should consider the occurrence of these more frequent channels or permutations of these channels.

With this set of data, it can be concluded that we cannot identify a single channel that is applicable to all subjects. In order to verify this conclusion, we sequentially input the indicator data corresponding to channels CP2, CP3, CP4, CP5, CP6, and TP7 into the model built above, and the results are consistent with our expectations. For all 41 subjects, the detection accuracy of a single channel is lower than that of the full channel. The detection results are very unstable. For some subjects, even though the accuracy of single-channel detection is lower than that of full-channel detection, it can still reach at least 0.8. However, for some subjects, the accuracy of single-channel detection is extremely low, at only 0.4. This conclusion suggests that, regardless of the channel, there will be cases in which the subjects are not applicable, which is due to the variability between individuals. It is not feasible to find a single channel that fits all 41 subjects.

In addition, regarding the situation in which the detection accuracy of a single channel is lower than that of a full channel, we speculate that this may be due to the fact that a single channel contains less information, which is insufficient to achieve the detection of the controller’s workload. In order to enhance the stability of detection, we considered increasing the number of channels. This not only augments the information within the channels but also reduces the variability among individuals. Accordingly, the six single channels described above were combined two by two to produce 15 channel combinations. The data corresponding to the 15 combinations were input into the model, and the accuracy of the model was output.

**Figure 16:**
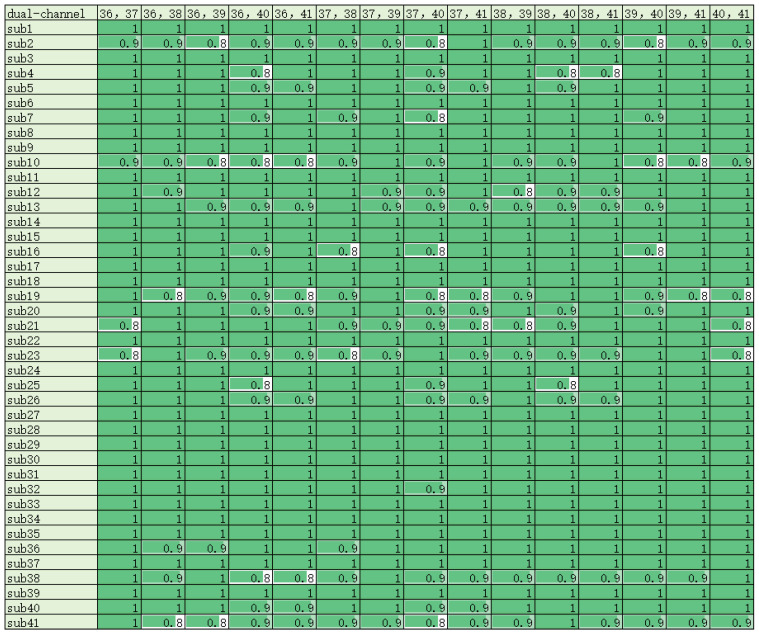
Results of the model based on dual channels.

The output results are shown Figure 16. It can be seen that the dual-channel system is much better compared to the single-channel system in terms of accuracy. Regardless of the channel combination, the accuracy for most subjects typically reaches up to 0.9. For some subjects, by excluding channels with lower correlations, the model’s accuracy can even reach 1. However, the dual-channel performance is unstable. Among the 15 channel combinations, there are instances where some subjects are not applicable. For instance, in the combination of channels CP2 and CP3, the detection accuracy for subjects 21 and 23 is only 0.8, which falls significantly below the required accuracy level. Each of the 15 channel combinations has between one and five subjects with low detection accuracy. In addition, the accuracy of 0.9 does not meet our practical needs.

**Figure 17:**
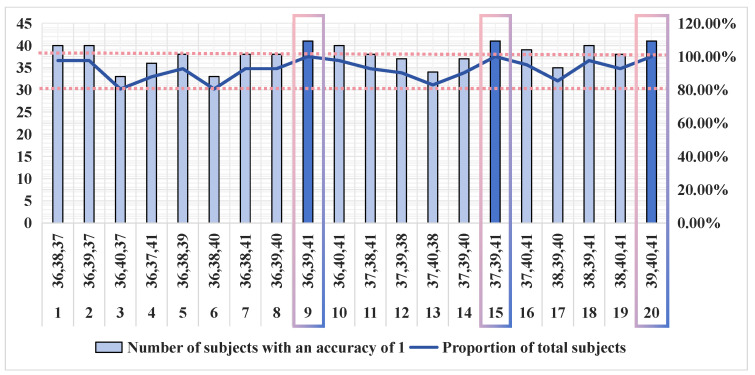
Statistical results of three-channel combinations.

Therefore, in order to enhance the accuracy and practicality of the model and eliminate situations where individual subjects are not applicable, increasing the number of channels was considered. This adjustment aimed to ensure that the detection accuracy remained stable across different subjects. In cases where the two-channel system failed to ensure stability, we utilized three-channel data to monitor the controller’s workload. This involved combining the aforementioned channels into various three-channel configurations, resulting in a total of 20 combinations. The corresponding three-channel data of 41 subjects were input into the model, and the results are shown in Figure 17.

As can be seen from the figure, the performance of the three-channel system is greatly improved compared to the two-channel system. Whether in terms of accuracy or stability, the performance of the three-channel combination is much better. As shown in the figure above, each channel combination includes more than 32 subjects whose detection accuracy can reach approximately 1. Nominally, each three-channel combination is optimal for over 80% of the subjects. For the remaining subjects whose detection accuracy does not reach 1, the detection accuracy can be as low as 0.8667. This result further validates the effectiveness of our channel filtering method based on the mRMR algorithm.

Among the 20 three-channel combinations, combinations 9, 15, and 20 performed the best and were the most stable in the model. For the 41 subjects involved in the experiment, the detection accuracy of these three combinations reached 1, and all other metrics were as expected. Therefore, we can conclude that the model based on three channels is applicable to all 41 subjects involved in this study. This implies that we can achieve the high-precision detection of controllers’ cognitive workload using only three channels. Meanwhile, for these 41 subjects, the three-channel model described in this paper can be directly applied in their work scenarios without considering individual differences. Only channels CP2, CP5, and TP7; channels CP3, CP5, and TP7; or channels CP5, CP6, and TP7 need to be processed when positioning the electrodes and EEG paste.

### 3.5. Results of Validation

According to the goals of the experiments, the model’s generalization ability in different subjects needed to be verified, so it was necessary to invite subjects besides those mentioned above to complete the experiment. In the verification experiment, four licensed controllers were invited to participate, with two male and two female controllers among the four. All participants provided informed consent before participating in the study and were able to proficiently operate the control simulation system.

**Figure 18:**
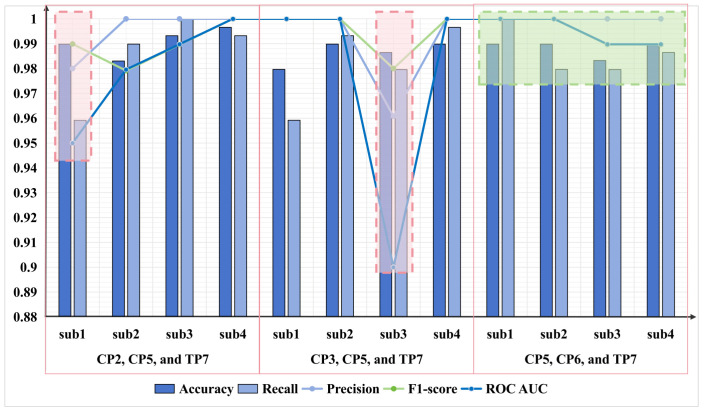
Validation results of the model.

Based on the previous results, the optimal features were found to be β + θ + α + γ, and the optimal channel combinations were channels CP2, CP5, and TP7; or channels CP3, CP5, and TP7; or channels CP5, CP6, and TP7. Therefore, the first step was to collect the EEG data of the controllers under different cognitive workloads. After preprocessing the data, the features of β + θ + α + γ were extracted from channels CP2, CP3, CP5, CP6, and TP7, and the data of the different channel combinations were input into the model based on the previous results. Then, the detection results for different subjects and channel combinations were output, as shown in Figure 18.

In regard to the three optimal channel combinations mentioned above, the verification experiment’s results showed a slight regression compared to the original results. The accuracy of all subjects in the original experiment could reach 1, but the detection accuracy in the validation experiment could reach 0.98 or higher. Among the other metrics, the F1 score was good, reaching 0.979 or higher in all channel combinations, while the precision and recall values in these three channel combinations could reach 0.96 or higher. However, the ROC AUC was somewhat poor, with a value of only 0.9 for sub3 in the channel combination of CP3, CP5, and TP7.

Comparing these three channel combinations, CP5, CP6, and TP7 showed better performance in all metrics and maintained robustness in detecting the cognitive workload in different controllers. Therefore, this channel combination demonstrated the highest universality and is most suitable for cognitive load detection in controllers.

## 4. Discussion

This study initially designed an experiment to obtain cognitive workload data from controllers; then, it delineated a series of features associated with gamma waves and proposed an indicator evaluation to identify the best-performing ones. Then, to enhance the accuracy and efficiency of cognitive workload detection, a workload detection method for controllers was developed and optimized from the perspective of channel reduction, making it more suitable for practical applications.

### 4.1. Proposal and Comparison of Indicators

As the indicators directly impact the accuracy of the detection work, it is necessary to initially propose indicators with better performance. In previous studies, EEG raw signals larger than 50 V are usually treated as artifacts [50,53], and there are fewer studies on gamma waves. In 2016, Thiago L.T. [23] detected drowsiness based on the spectral power indicators γ/δ and (γ + β)/(δ + α) and achieved better results. However, there are fewer studies that consider high-frequency gamma waves in the selection of fatigue and workload indicators. As gamma waves have been shown to be related to work and memory [25], it is logical to suppose that utilizing indicators associated with gamma waves could be used for workload detection.

Therefore, in order to fill the research gaps in this area and identify suitable workload detection indicators for controllers, this study first proposed a set of power spectral indicators. These include 13 common workload detection indicators related to the δ wave, α wave, β wave, and θ wave, as well as 13 new indicators associated with gamma waves. After proposing and evaluating the indicators, we discovered that, among the 26 indicators, the two best-performing ones were associated with gamma waves. Furthermore, in assessing the absolute energy of the five bands (γ > β > α > θ > δ), the findings of this study were consistent with the previous literature on the absolute energy of the delta, theta, alpha, and beta waves [49]. This also demonstrates the reliability of the indicator evaluation approach proposed in this paper.

In addition, other conclusions could be derived from the study’s findings. The evaluation result of the indicator β + θ + α + γ was better than that of γ > β > α > θ > δ. The addition of δ led to a decline in the indicator’s performance. It is worth noting that the δ wave is not suitable for the detection of the cognitive workload. Consistently, research indicates that the delta wave is prominent during mental trance or hypnotic states in individuals [54,55,56,57], rather than during the waking state of a controller at work. Moreover, compared with the indicator δ + β + θ + α, the evaluation result of δ + β + θ + α + γ is better, indicating that the addition of γ enhances the performance of the indicator. This is also consistent with our expectation that the indicator related to gamma waves can be used for workload detection. Meanwhile, based on this conclusion, future research can commence by selecting indicators related to gamma waves, exploring their mechanism in detecting the cognitive workload, and investigating additional relevant and high-precision indicators.

### 4.2. Evaluation Method for Indicators and Its Application

After proposing the indicators, it is necessary to compare them to determine which one is most suitable for the corresponding testing work. Li Wei [29] calculated 12 energy indicators based on EEG data. Gray relational analysis (GRA) was then introduced to determine the most effective indicators of driver fatigue. This method is able to comprehensively consider the effects of multiple factors and has a certain degree of robustness to incomplete, inaccurate, or noisy data. However, it needs to be applied after data normalization, which increases its computational complexity. In addition, gray correlation analysis requires the determination of the weighting parameters and the calculation of the gray correlation, which involve subjective judgment. The calculation results may have a degree of subjectivity, potentially impacting the final outcomes.

Thiago L. T. [23] evaluated the validity of γ/δ and (γ + β)/(δ + α) in detecting sleepiness by comparing them with five multichannel indicators studied by previous researchers. The Wilcoxon signed-rank test was utilized to assess the performance of the indicator. This method shares similarities with the approach discussed in this paper, which involves comparing new indicators with existing ones to confirm their reliability. However, this method only determines the quality of the indicators based on whether they exhibit significant changes or not; it does not allow for a quantitative evaluation of the indicators.

In order to better evaluate the performance of the indicators, we designed an indicator evaluation method based on the mRMR algorithm. This algorithm has been utilized to select a subset of features from EEG data with improved performance [40]. In this paper, we discuss the mRMR algorithm’s approach to calculating the correlation between the target variable and the set of features, as well as its method of quantifying the redundancy among the features. We also examine how it measures the correlations between the indicators and cognitive workload, along with the redundancy across all channels. Compared with existing techniques, the feature evaluation technique based on the mRMR algorithm proposed in this paper avoids the subjectivity of the gray correlation analysis method in determining the weight parameters and gray correlation and the classifier setting parameters. It avoids the discrepancies of the classifier evaluation method and it considers the redundancy of the internal information of the channel compared with existing feature selection algorithms, so that the evaluation result is more accurate. Compared to the related techniques based on statistics, a quantitative evaluation of the performance of different features can be performed so that they can be compared.

Although the mRMR algorithm has many advantages, it also has certain limitations. This algorithm has high computational complexity and may not be efficient for large datasets. Specifically, during the calculation of mutual information, the mRMR algorithm requires the extensive computation of the probability density and multivariate probability density, resulting in relatively high computational complexity. For large datasets, this computational complexity can become a bottleneck in terms of efficiency, causing the algorithm to perform poorly when handling massive data volumes.

In addition to being used in the evaluation of cognitive load-related indexes, the feature evaluation method can also be applied in a variety of other fields, such as fatigue level analysis, emotion recognition, attention monitoring, concentration assessment, working memory assessment, anxiety and stress assessment, etc., depending on the target variables. The specific steps of use are as follows: (1) set the target variable; (2) obtain the EEG data of the subject at different levels of the target variable; (3) extract the EEG features; (4) calculate the sum of the mutual information between all channels and the target variable and the sum of the redundancy within the channels under different EEG features; (5) find the evaluation metrics corresponding to all features and rank them. By calculating the sum of the mutual information between all channels and the target variables and the sum of the redundancy within channels, we can determine the values of the evaluation metrics corresponding to the features and then sort them to find the best features.

### 4.3. Screening Methods for EEG Channels

A large number of EEG channels can significantly impact the method’s application and popularity. Therefore, it is essential to screen the channels to retain the effective ones and eliminate the useless ones. Li Wei [29] used kernel principal component analysis (KPCA) to reduce the number of electrodes and established a driver fatigue evaluation model using regression equations based on the EEG data from two significant electrodes (Fp1 and O1). In kernel principal component analysis (KPCA), it is crucial to select a suitable kernel function and its related parameters. Different kernel functions and parameter choices result in varying downscaling effects, but there is no universal method to determine the optimal parameters. It needs to be debugged experimentally and empirically. Min [37] proposed a simplified method for electrode selection to calculate the weights of each electrode based on the accuracy in order to identify the most important electrodes. This method can only identify two significant electrodes, which may become ineffective when the accuracy of two-channel detection does not meet the requirements.

In order to solve this problem, we introduce the mRMR algorithm for the selection of EEG channels. This method avoids the need to select kernel functions and parameters, and it also enables the number of channels to be chosen arbitrarily based on the demand. Moreover, the channel combinations selected by this algorithm have a strong correlation with the workload and are relatively independent of each other. Based on the results of the model’s optimization, it is known that the EEG channels selected using this algorithm can be used for the cognitive workload detection of controllers.

### 4.4. Model Based on Full Channels

In this study, a series of indicators related to gamma waves were proposed. The mRMR algorithm was used to filter the indicators that were more sensitive to the workload. Subsequently, a full-channel controller workload detection model was established based on the indicators. In the full-channel model, for our 41 subjects, the model’s accuracy ranged from 0.979 to 0.996. Although the ROC AUC value for subject 33 was not as high as 0.889, overall, the results of the full-channel model were as expected and it could be utilized to detect the workloads of controllers. Since there are not many existing workload detection models for controllers, this study uses a fatigue detection model for comparison. Nan Wu et al. [58] proposed a speech-based fatigue state detection method that combines the quantum genetic algorithm with an adaptive strategy. The detection accuracy of this method is up to 98.5%. Zhang et al. [59] extracted the wavelet entropy and spectral entropy (SE) of EEG, as well as the wavelet entropy of EOG and amplitude entropy (AE) of EMG, to estimate the level of driving fatigue. The accuracy achieved ranged from 96.5% to 99.5%. Compared with the existing models, this study achieved a certain breakthrough in accuracy. In addition to the influence of the method, another reason for this may be that EEG data are more accurate compared to speech data and EMG data [60].

### 4.5. Model Based on Fewer Channels

On the basis of screening sensitive indicators, the mRMR algorithm was used to identify sensitive channels. A three-channel model was established, and, after eliminating unnecessary and redundant channels, the model’s accuracy was significantly improved, reaching an accuracy rate of 1. Among the existing fewer-channel models, Fu et al. [61] proposed a fatigue detection model based on the Hidden Markov Model and the fusion of physiological and situational knowledge to assess the probability of fatigue. Based on EEG signals from two channels (O1 and O2) and other physiological signals, the highest accuracy achieved was 92.5%. Li et al. [29] collected EEG data from 16 channels and calculated 12 energy parameters. The number of electrodes was reduced using kernel principal component analysis (KPCA). Two channels (FP1 and O1) showed the highest accuracy of 91.5% in the experimental results. Xiong et al. [62] combined acoustic emission (AE) and surface electromyography (SE) features with a support vector machine (SVM) classifier to detect driver fatigue, achieving the highest accuracy of 91.3% in the P3 channel. Jianfeng Hu et al. [32] used entropy measurements to extract features from individual electroencephalogram (EEG) channels. The sample entropy (SE), fuzzy sample entropy (FE), fuzzy entropy (AE), approximate entropy, and spectral entropy (PE) were utilized for the analysis of raw EEG signals and compared with 10 classifiers to optimize the performance for a single channel. The highest accuracy of 96.6% was achieved. This comparison shows that the method proposed in this paper, although slightly misaligned with the current technology in terms of the number of channels, has achieved a significant breakthrough regarding the accuracy of models with fewer channels. This breakthrough means that it successfully retains or even exceeds the accuracy of multichannel detection while reducing the number of channels.

### 4.6. Influence of Number of Channels on Detection Accuracy

**Figure 19:**
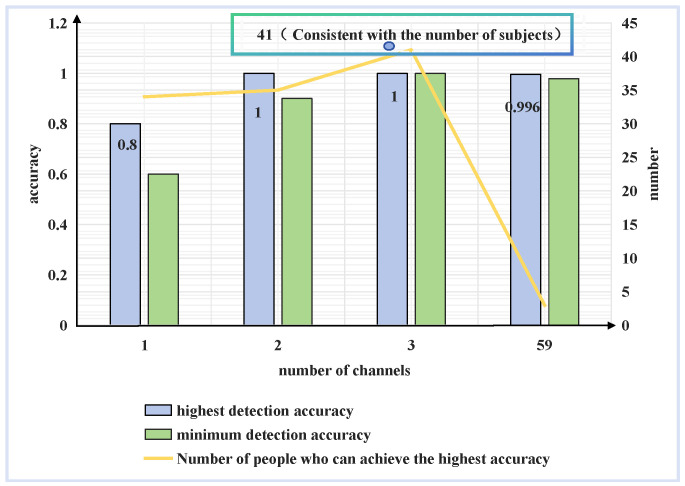
Results of some of the best channel combinations.

Single-channel data, two-channel data, and three-channel data were used for the detection of the controllers’ workload. The detection results of some of the best channel combinations are shown in Figure 19. Accordingly, the impact of the number of channels will be analyzed in terms of the detection accuracy, detection stability, and practical usability.

#### 4.6.1. Detection Accuracy

The impact of the number of channels on the accuracy is typically analyzed by comparing the highest detection accuracy. The higher the accuracy, the more effective the screened channels are. In the detection results of 41 subjects, the highest detection accuracy for a single channel is up to 0.8, and the highest detection accuracy for dual and triple channels is up to 1. For the indicator β + θ + α + γ, the accuracy of the single best channel is much lower than that of the full channel. This difference may be attributed to the insufficient amount of information about this indicator contained in the single channel, hindering the detection of the cognitive workload. The highest accuracy for the dual and triple channels is higher than that of the full channel at 1, which indicates that the elimination of channels can lead to an increase in accuracy. This further suggests that the eliminated channels are redundant or ineffective. Therefore, it can be concluded that the screening of channels with the mRMR algorithm is effective.

#### 4.6.2. Detection Stability

The impact of the number of channels on the detection stability can be analyzed by comparing the minimum detection accuracy and the number of individuals in whom the highest accuracy can be achieved. The higher the minimum accuracy, the more stable the screened channels obtained for all subjects, making them more suitable for workload detection. In this study, the minimum detection accuracy of a single channel is only 0.4, which indicates that a single channel alone cannot achieve the detection of the cognitive workload of all 41 subjects. The minimum detection accuracy of a two-channel combination is 0.8–0.9. After incorporating the data from an additional channel, the minimum detection accuracy is significantly increased. However, this level of accuracy is still insufficient in meeting the requirements for practical detection tasks. Among the 20 three-channel combinations, some of them achieve minimum detection accuracy of 0.87–0.93, showing significant improvements in stability. The optimal channel combination achieves detection accuracy of 1 for all subjects, suggesting that this channel combination is sufficiently stable for practical detection tasks. In this work, the need for detection stability may be attributed to the large number of subjects, necessitating three-channel detection. Conversely, if the number of subjects is small, two channels may suffice to ensure stable detection.

#### 4.6.3. Practical Usability

From the above analyses, we can conclude that in actual detection work, the optimal number of channels is not necessarily the smallest or the largest. A smaller number may result in unstable detection performance, while a larger number of channels may lead to redundancy and inconvenience in detection work. In applications, the number of channels may need to be adjusted according to the actual situation. For instance, when the number of subjects is small, two channels may be adequate for the application. However, if the number of subjects is large, the stability of three channels may not be sufficient for the high-precision detection of all subjects. In such cases, it may be necessary to consider increasing the number of channels.

### 4.7. Methods to Deal with Individual Variability

In addition to the mentioned conclusions, this study also found that when using the mRMR algorithm to select channel combinations, the optimal channel combinations for individual subjects were not entirely consistent. This inconsistency was caused by the differences between the individuals. Due to humans having limited cognitive resources and attention abilities, even if two different individuals complete the same tasks, their cognitive workloads in processing such tasks may still differ [36]. Therefore, the applicability of the results to different individuals needs to be considered. To address the fact that the optimal channel combinations vary from person to person, this study first identified the most frequently occurring channels and organized them into different combinations. By exploring these combinations, the channel combination most suitable for this group of subjects can be selected.

In practical applications, flexibility can be exercised based on specific circumstances. For example, when the number of controllers is small, the EEG data of each controller can be input into the mRMR algorithm to determine the optimal channel for each controller. This approach can create a single channel for different individuals, enabling the more accurate and efficient detection of all controllers. In situations with a larger number of controllers, as discussed in this study, we can filter out the channel combinations that are most suitable for all controllers. If the accuracy and stability of single or dual channels are insufficient, increasing the number of channels can be considered, establishing three-channel or four-channel models.

In terms of theory, the research results presented in this paper can provide a basis for EEG indicator screening and EEG channel screening and enhance the theoretical framework related to the workload detection of controllers. In practical terms, the model proposed in this paper reduces the number of channels while maintaining accuracy. This significantly decreases the complexity of applying workload detection via EEG and enables the more efficient and precise detection of the controllers’ workloads. However, there are still deficiencies, as pointed out in this paper. Additionally, although the usability of indicator 26 has been verified, the model parameter settings are tailored to the existing subjects. Whether they are applicable to other subjects still needs to be verified. In the channel-less model, it remains to be verified whether the channel combination is applicable to other subjects.

## 5. Conclusions

In this paper, we propose an EEG indicator evaluation method based on the mRMR algorithm and establish a cognitive workload detection model for controllers based on the indicators selected through this method. Afterwards, the mRMR algorithm is used to screen the channel combinations applicable to 41 subjects, resulting in a reduction in channels while maintaining the accuracy of detection.

From the perspective of practicality and the methodology, the current study makes the following three contributions.

(1) Indicators related to gamma waves were proposed, and the validity of some of them was verified through different methods.
(2) An EEG feature evaluation method based on the mRMR algorithm was proposed and verified. This method allows for the quantification of the correlation between each channel and the cognitive workload, as well as the redundancy between different channels.
(3) A cognitive workload detection method based on three channels for controllers was constructed, and cognitive workload detection for 41 subjects was achieved using only three channels.

It should also be noted that while this study achieved good results using the mRMR algorithm, this algorithm has high computational complexity, which may lead to reduced efficiency when handling large datasets. Therefore, future research should also focus on optimizing this algorithm to ease the calculation process.

## 6. Patents

Patents CN118078288A and CN118078287A resulted from the work reported in this manuscript.

## Figures and Tables

**Table 1:** Scenario settings.

Scenario	Phase	Air Traffic Flow
Scenario 1	Baseline	6
Scenario 2	High-Workload Nominal	21
Scenario 3	Overload Nominal	30
Scenario 4	High-Workload Off-Nominal	21

**Table 2:** Selection of indicators.

Indicator Number	Name	Indicator Number	Name
Indicator 1	δ	Indicator 14	γ absolute energy
Indicator 2	θ	Indicator 15	δ + β + θ + α + γ
Indicator 3	α	Indicator 16	β + θ + α + γ
Indicator 4	β	Indicator 17	γ/δ
Indicator 5	δ + β + θ + α	Indicator 18	γ/θ
Indicator 6	δ/(δ + β + θ + α)	Indicator 19	γ/α
Indicator 7	θ/(δ + β + θ + α)	Indicator 20	γ/β
Indicator 8	α/(δ + β + θ + α)	Indicator 21	γ/(α + β)
Indicator 9	β/(δ + β + θ + α)	Indicator 22	γ/(θ + β)
Indicator 10	θ/β	Indicator 23	γ/(θ + α)
Indicator 11	α/β	Indicator 24	γ/(θ + β + α)
Indicator 12	(α + θ)/β	Indicator 25	γ/(δ + β + θ + α)
Indicator 13	(α + θ)/(α + β)	Indicator 26	γ/(δ + β + θ + α + γ)

**Table 3:** Evaluation results of indicators.

Indicator	Φ	Indicator	Φ	Indicator	Φ
Indicator 16	3688.969054	Indicator 10	1459.994293	Indicator 18	149.8918293
Indicator 15	3615.334366	Indicator 11	1252.504293	Indicator 6	25.13292683
Indicator 5	3585.256829	Indicator 13	1426.177537	Indicator 26	2.167195122
Indicator 4	3194.823902	Indicator 20	931.4538049	Indicator 7	0
Indicator 14	3178.441195	Indicator 19	809.6299756	Indicator 8	0
Indicator 3	2970.58939	Indicator 21	600.6179512	Indicator 9	0
Indicator 2	2882.96239	Indicator 22	312.6046341	Indicator 17	0
Indicator 1	2797.489585	Indicator 24	246.5156098	Indicator 25	0
Indicator 12	2083.364463	Indicator 23	154.1476829		

## Data Availability

The data presented in this study are available on request from the corresponding author. The data are not publicly available due to confidentiality issues.
